# PERCEIVED STRAIN ON THE SPOUSAL RELATIONSHIP AMONG MARRIED CUSTODIAL GRANDPARENTS WITHIN THE PARENTING ROLE

**DOI:** 10.1093/geroni/igad104.0956

**Published:** 2023-12-21

**Authors:** Eunbea Kim, Jeong Eun Lee, Gregory Smith

**Affiliations:** Iowa State University, Ames, Iowa, United States; Iowa State University, Ames, Iowa, United States; Kent State University, Kent, Ohio, United States

## Abstract

Providing primary care for grandchildren is known to have a significant impact on custodial grandparents’ well-being. For married custodial grandparents, although their caregiving burden may spill over to marriage which can lead to negative psychological outcomes, it has rarely been examined. We examined how custodial grandparents couples’ perceived stress in the spousal relationship due to parenting (PSSR) is associated with their psychological outcomes mutually using Actor Partner Interdependent Model. We further tested the moderating effect of coping strategies on the association between PSSR and psychological outcomes. Participants are grandparents couples providing full-time care to their grandchild in the absence of the grandchildren’s biological parents (N_dyad = 193). We found that one’s PSSR was associated with one’s higher depressive symptoms for both grandmothers and grandfathers. Regarding anxiety symptoms, only grandmothers’ PSSR was positively related to both their own and their spouses’ anxiety level.In terms of coping strategies, we found that a higher level of problem-focused coping was related to higher levels of anxiety for both grandmothers and grandfathers when grandmothers experienced higher PSSR. A higher level of emotional coping was related to higher level of depressive symptoms in both grandfathers and grandmothers when they experienced a higher level of PSSR. A higher level of emotional coping was related to both grandfathers and grandmothers’ higher level of anxiety when grandmother’s PSSR was higher. Our research adds to the existing body of literature by emphasizing how emotional and problem-focused coping can moderate the connection between increased PSSR and psychological outcomes among custodial grandparents.

